# Unexpected Absence of Skeletal Responses to Dietary Magnesium Depletion: Basis for Future Perspectives?

**DOI:** 10.3390/biomedicines11030655

**Published:** 2023-02-21

**Authors:** Marzia Ferretti, Francesco Cavani, Vincenza Rita Lo Vasco, Marta Checchi, Serena Truocchio, Pierpaola Davalli, Chiara Frassineti, Federica Rizzi, Carla Palumbo

**Affiliations:** 1 Department of Biomedical, Metabolic and Neural Sciences, University of Modena and Reggio Emilia, 41125 Modena, Italy; 2 Biomedical and Neuromotor Sciences, DIBINEM Alma Mater Studiorum, University of Bologna, 40126 Bologna, Italy; 3 Department of Medicine and Surgery, University of Parma, 43125 Parma, Italy

**Keywords:** Mg-deficient diet, osteoporosis, C57BL/6J mice, bone histomorphometry, gene expression

## Abstract

It’s known that a magnesium (Mg)-deficient diet is associated with an increased risk of osteoporosis. The aim of this work is to investigate, by a histological approach, the effects of a Mg-deprived diet on the bone of 8-weeks-old C57BL/6J male mice. Treated and control mice were supplied with a Mg-deprived or normal diet for 8 weeks, respectively. Body weight, serum Mg concentration, expression of kidney magnesiotropic genes, and histomorphometry on L5 vertebrae, femurs, and tibiae were evaluated. Body weight gain and serum Mg concentration were significantly reduced, while a trend toward increase was found in gene expression in mice receiving the Mg-deficient diet, suggesting the onset of an adaptive response to Mg depletion. Histomorphometric parameters on the amount of trabecular and cortical bone, number of osteoclasts, and thickness of the growth plate in femoral distal and tibial proximal metaphyses did not differ between groups; these findings partially differ from most data present in the literature showing that animals fed a Mg-deprived diet develop bone loss and may be only in part explained by differences among the experimental protocols. However, the unexpected findings we recorded on bones could be attributed to genetic differences that may have developed after multiple generations of inbreeding.

## 1. Introduction

Magnesium (Mg) is the fourth most abundant cation in the body and the second most widespread intracellular one [1,2,3]. Overall, the predominant action of Mg is related to ATP utilization; in fact, it exists in all cells mainly as Mg-ATP. Regarding its physiological role, Mg is essential for many enzymatic reactions by stabilizing the reaction intermediate and/or the output product and simultaneously binding two substrates for the purpose of facilitating the reaction [4,5]. A number of signaling cascades related to Mg involve protein kinases, such as lymphocyte-specific protein tyrosine kinase (Lck), zeta-chain-associated protein kinase 70 (ZAP-70), and interleukin-2-inducible T-cell kinase (Itk) [6]. Approximately 60–65% of the total magnesium is present in the skeleton embedded in hydroxyapatite crystals, 32–35% is complexed with proteins and nucleic acids, and only 1–2% in plasma and other minor forms of deposition [4,7,8].

In humans, dietary Mg deficiency has been associated with hypertension, cardiac arrhythmias, myocardial infarction, hypokalemia, hypocalcemia, and the risk of osteoporosis; in fact, in disorders that impair intestinal absorption of Mg and/or are associated with renal loss of Mg (such as malabsorption syndromes, alcoholism, diabetes mellitus, and drugs such as diuretics), the incidence of osteoporosis is higher than expected [9]. Several experimental studies in rodents have shown that progressively reduced Mg diets, compared with the recommended daily intake, result in a corresponding loss of bone mass and increased skeletal fragility, due to increased bone resorption and/or reduced bone formation during skeletal remodeling [10,11,12,13,14,15]. In fact, Mg has been shown to be important for bone cell activity: (i) in vitro its depletion causes inhibition of osteoblastic growth, while its presence has a mitogenic action [16,17]; (ii) in vivo, its depletion causes both a decrease in the number of osteoblasts associated with alteration of their function and an increase in the number of osteoclasts and the surfaces eroded by them [11,12,13,18,19]. In addition, magnesium deficiency increases the production of pro-inflammatory cytokines, such as *tumour necrosis factor-α* (*TNF-α*), *interleukin-1* (*IL-1*), and *substance P*, which promote bone resorption by osteoclasts [13].

Among rodents, the C57BL/6 mouse is widely used in biomedical research in studies of effects related to dietary magnesium reduction on physiological processes in various tissues/organs, as blood cells, liver, and the brain [20,21,22,23,24]. From the founding line C57BL/6, after many generations of inbreeding, several substrains have been derived that differ in small genomic variations leading to different phenotypes; of these, the most widely used in research are C57BL/6N and C57BL/6J [25,26]. As for bone, Simon and coworkers [26] have shown that, under normal conditions, the overall bone trabecular network is similar between the two substrains, as are bone formation and resorption markers. Only Tu and coworkers [27] investigated the effect of the reduction of magnesium in the diet for 8 weeks in C57BL/6J male mice using a radiologic and biochemical approach; they found a reduction of bone volume, number of trabecular segments, and bone mineral content of the femoral distal metaphysis and a decrease in Mg in plasma. 

The purpose of the present work is to investigate with a histological approach the effects of a Mg-deprived diet on C57BL/6J mice to better understand the influence of this diet on bone architectures, in particular of the axial *versus* appendicular skeleton.

## 2. Materials and Methods

### 2.1. Experimental Animals and Treatment

8-week-old C57Bl/6 male mice (n = 14) were obtained from the animal facility of the University of Parma. To minimize genetic drift from the founder line, the animals are back-crossed with C57Bl6/J pure strain mice purchased from Charles River (Charles River Laboratories, Calco, ITA) every 10 generations. The animals of the colony exhibit a very good degree of uniformity in terms of reproductive capacity, rate of growth of newborns, body weight, general appearance, and behavior.

All mice were supplied with a basal diet over a one-week acclimatization period. Subsequently, the mice were randomized into two groups, each containing seven mice: the treatment and control groups. In the treatment group, mice were fed a commercial Mg-deprived diet (0% Mg wt/wt, Charles River Laboratory, Calco, ITA) and distilled water ad libitum for 8 weeks; in the control group, mice were fed a normal diet (0.2% Mg wt/wt, Mucedola, Settimo Milanese, ITA) and natural tap water ad libitum for 8 weeks. A complete nutritional description of the two diets is supplied in Table S1 (Supplementary Materials). Equal appreciation of the two diets was assessed by weighting daily the amount of food consumed by control and treated mice. All animals were maintained in a temperature-controlled room (22 ± 1 °C) with a 12:12 h light:dark cycle. The body weight of each animal was recorded at the beginning of the experimental period and then every 14 days until the day of sacrifice.

After 8 weeks, all mice were anesthetized with isoflurane and blood samples were collected by cardiac puncture; then, mice were euthanized by cervical dislocation. Kidneys and bones were collected for subsequent biochemical, histological, and histomorphometrical analyses. Blood samples were allowed to clot for 30 min and then centrifuged at 10,000 rpm for 10 min at room temperature. Serum was separated from the clot and stored at −20 °C until use. For 1 control and 1 treated animal, serum couldn’t be properly separated from blood.

All experiments conformed to the National Research Guide for the Care and Use of Laboratory Animals (D.L. 26/2014) and the recommendations of the Institutional Animal Care and Use of the University of Parma (identification code Project ID: 719/2015-PR). All achievements were made to minimize animal suffering and reduce the number of animals used. 

### 2.2. Serum Mg Concentration

Mg concentration was assessed in serum samples by using a specific commercial colorimetric enzyme (glycerol kinase) assay kit (Sigma Aldrich, St. Louis, MO, USA). The assay was performed according to the manufacturer’s instructions. Briefly, 5 µL of each serum sample was directly pipetted into a 96-well plate and added with 50 µL of Master Reaction Mix (35 µL Magnesium Assay Buffer, 10 µL Developer 5 µL Magnesium Enzyme Mix), mixed on a horizontal shaker, and incubated for 10 min at 37 °C. The absorbance at 450 nm was measured immediately after the incubation period (A_450_)_initial_ and then every 5 min until the highest measure approached the value of 1.5 (A_450_)_final_. For each sample (A_450_)_final_ − (A_450_)_initial_ was calculated to correct for the non-specific background of *nicotinamide adenine dinucleotide phosphate* (NADPH) present in the sample. The Mg concentrations of unknown samples were determined by comparison with a calibration curve obtained from Mg standard solution at 0; 0.06; 0.08; 0.12; 0.18; 0.24; 0.30 nmol/µL.

### 2.3. RNA Extraction, cDNA Preparation, and qPCR

Kidney tissue samples obtained from normal or Mg-deprived diet-fed mice were snap-frozen in liquid nitrogen and ground to a fine powder. RNA was extracted starting from 30 mg of powder by using 1 mL of TRIZOL reagent (Thermo Fisher Scientific, Waltham, MA, USA) and then purified with the PureLink RNA Mini kit (Thermo Fisher Scientific, Waltham, MA, USA). cDNA Synthesis was carried out with the RevertAid First Strand cDNA Synthesis kit (Thermo Fisher Scientific, Waltham, MA, USA) according to the manufacturer’s instructions using a previously optimized protocol [28]. The expression of *magnesium transporter protein 1 (MagT1), transient receptor potential cation channel, subfamily M, member 6 (TRPM6), transient receptor potential cation channel, subfamily M, member 7 (TRPM7)* was measured by qPCR amplification using 1 µL of cDNA with the SSOAdvanced Universal SYBR Green Supermix (Bio-Rad, Hercules, CA, USA) on the DNA Engine Opticon 4 (MJ Research, Walthman, MA, USA), as previously extensively described [28]. The sequence of the primers used in qPCR is reported in Table 1, *glyceraldehyde-3-phosphate dehydrogenase (GAPDH)* was used as the housekeeper gene. For each analysis, the samples were run in duplicate. The gene expression level was calculated by the 2^−∆∆Ct^ method, where Ct = threshold cycle; ∆Ct = Ct *(target gene*) − Ct *(GAPDH)* and ∆∆Ct = ∆Ct_target gene_ Treated − ∆Ct_target gene_ Control;

### 2.4. Histology and Histomorphometry

Soon after sacrifice, the fifth lumbar vertebra (L5), the femurs, and the tibiae from each animal were retrieved, cleaned of soft tissues, and fixed in a solution of 4% paraformaldehyde with a pH of 7.4 in sodium phosphate saline (PBS).

#### 2.4.1. Resin-Embedded Specimens

Vertebrae, right femurs, and right tibiae were then dehydrated in increasing ethanol concentrations and embedded in methyl-methacrylate (Sigma Aldrich, Milan, Italy). Specimens were cut with a Leica SP 1600 diamond saw microtome (Leica SpA, Milan, Italy) to obtain transverse serial sections of 200 µm thickness. The sections, taken from the center of L5, from the middle of the femoral patellar groove, and just below the tibial condyles, were glued to a glass slide and ground to a final thickness of 40 µm; they were then superficially stained with Fast green 1% + 1% acetic acid, observed with a Nikon Eclipse Ni microscope equipped with a DS-Fi2 camera (Nikon, Tokyo, Japan) and images were acquired by means of NIS-Elements D 5.11.00 software (Nikon, Tokyo, Japan). Histomorphometry was performed by means of the software Image J (NIH, Bethesda, Maryland, USA) to calculate:

- trabecular bone volume (BV/TV), trabecular thickness (Tb.Th), trabecular number (Tb.N), and trabecular separation (Tb.Sp) in the L5 body, femoral distal, and tibial proximal metaphyses;
- anterolateral cortical thickness (Ct.Th) measuring it at 10 random points in the vertebral body.

All measurements were performed according to the American Society for Bone and Mineral Research (ASBMR) histomorphometry nomenclature [29].

#### 2.4.2. Paraffin-Embedded Specimens

Left femurs and left tibiae were decalcified in a 10% EDTA solution for 20 days, then were washed with PBS, dehydrated in graded ethanol, and embedded in paraffin. Longitudinal serial sections (5 µm thick) of the femoral distal metaphysis and tibial proximal metaphysis were stained with Toluidine blue & Eosin to measure at 10 random points the thickness of the growth plate. Tartrate Resistant Acid Phosphatase (TRAP) reaction was used to identify osteoclasts; the osteoclasts number (NOc) was calculated along the bone surface (BS) in the interface along the growth cartilage and diaphyseal bone and expressed as NOc/BS. All slides were observed with a Nikon Eclipse Ni microscope equipped with a DS-Fi2 camera (Nikon, Tokyo, Japan) and evaluated by means of NIS-Elements D 5.11.00 software (Nikon, Tokyo, Japan).

### 2.5. Statistical Analysis 

The IBM SPSS v25.0 statistical package (International Business Machines Corporation, Armonk, NY, USA) was used. Data are expressed as mean values ± standard deviation of the mean (SD). The statistical analysis was based on the Student’s *t*-test. The limit of statistical significance was set at *p* < 0,05. Graphics were prepared by using the GraphPad Prism v9.0 software (GraphPad software, San Diego, CA, USA).

## 3. Results

### 3.1. Effects of the Mg-Deprived Diet on Body Weight and Mg Serum Concentration 

**Figure 1:**
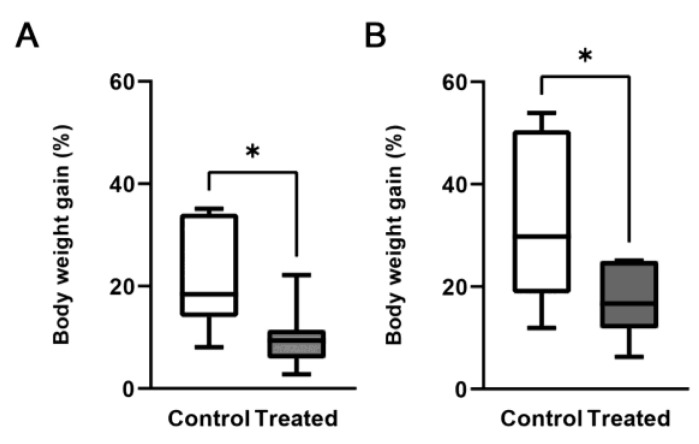
Body weight gain. Mice were fed either a normal (control) or a Mg-deficient diet (treated). Body weight gain was calculated after 4 weeks (**A**) and 8 weeks (**B**) as % of the initial value. Data (n = 7 for each group) are presented as, box-and-whisker plots- (Center line, median; box limits, first and third quartiles; whiskers, max-min). * Significant differences of the treated group vs. the control group (*p* < 0.05).

All the animals included in the study gained weight during the 8-week study. However, the body weight gain was significantly reduced to 50% after 4 weeks and to 45% after 8 weeks in mice receiving the Mg-deprived diet compared with mice receiving the control diet (Figure 1).

**Figure 2:**
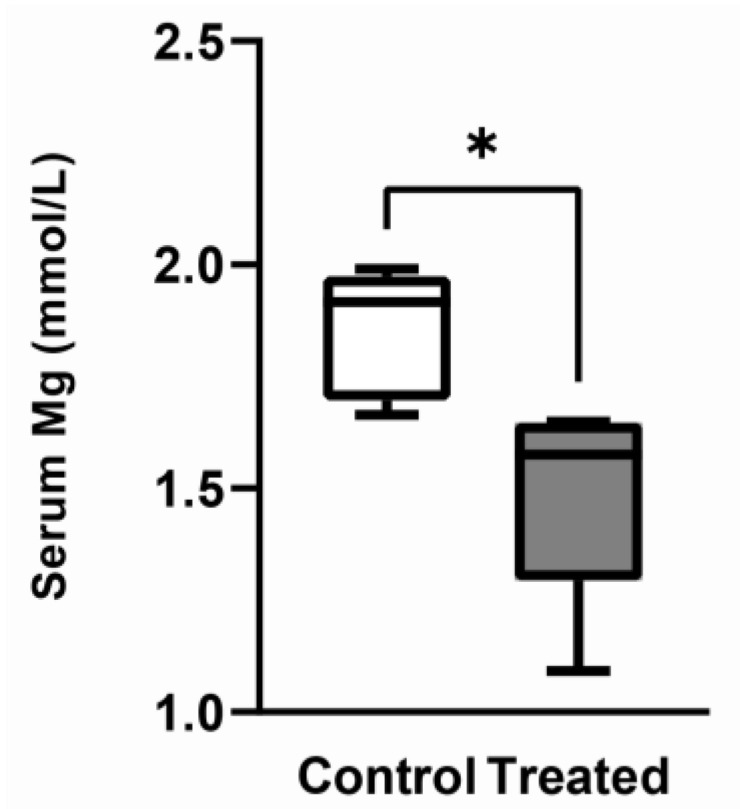
Serum Mg concentration. Mg was measured in serum of mice fed either a normal (control) or a Mg deficient (treated) diet. Data (n = 6 per each group) are presented as box-and-whisker plots- (Center line, median; box limits, first and third quartiles; whiskers, max-min). * Significant differences of the treated group vs. the control group (*p* < 0.01).

After 8 weeks of treatment, mice fed the Mg-deficient diet exhibited a significant reduction (20%) in serum Mg concentration compared with the control group (Figure 2).

### 3.2. Expression of TRPM6, TRPM7 and MagT1

**Figure 3:**
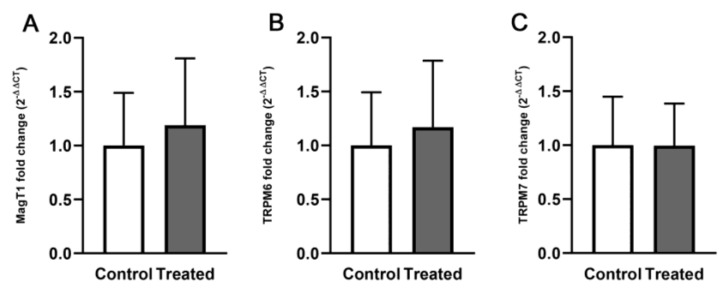
Relative expression of Mg transporters mRNA levels. mRNA was extracted from kidney tissue of mice fed either a normal (control) or a Mg deficient diet (treated) for eight weeks, retro transcribed and amplified by q-PCR. Data (n = 7 for each group) are presented as a bar graph. The relative fold change of *MagT1* (**A**), *TRPM6* (**B**) and *TRPM7* (**C**) mRNA of treated samples vs. controls has been calculated via the 2−∆∆Ct method. GAPDH was used as housekeeping gene; Ct = threshold cycle, ∆Ct = normalized Ct = Ct(*Target*) − Ct(*GAPDH*) and ΔΔCt = ∆Ct*Target*(treated sample) − ∆Ct*Target*(control sample).

By quantitative PCR (qPCR), we investigated whether animals fed with the Mg-deprived diet for 8 weeks exhibited differences in the expression of Mg transport and homeostasis systems compared with controls. To this end, we measured the relative expression levels of *MagT1, TRPM6,* and *TRPM7* mRNA in frozen kidney tissue samples. Chronic Mg deprivation did not have any significant effect on the expression of Mg transporters in the kidney, as reported in Figure 3.

### 3.3. Histology and Histomorphometry

**Figure 4:**
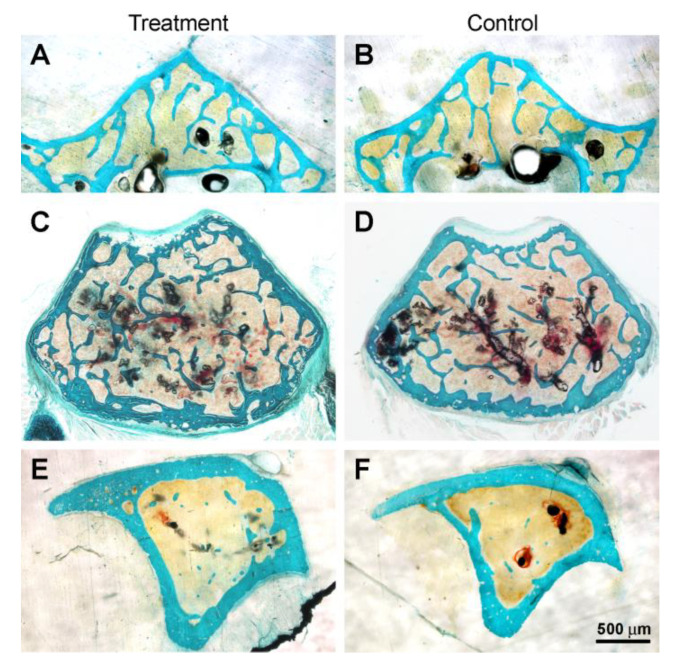
Micrographs of representative histological transverse sections of L5 vertebral body (**A**,**B**), femoral distal metaphysis (**C**,**D**), and tibial proximal metaphysis (**E**,**F**) of treatment and control groups.

Unexpectedly, the histological observations of all bone segments investigated showed that their morphological aspect is very similar in both the treatment (Mg-deprived diet) and control groups (Figure 4). Histomorphometric data are reported in Table 2; no significant differences were found between the experimental groups for all the parameters evaluated, thus confirming the histological observations.

**Figure 5:**
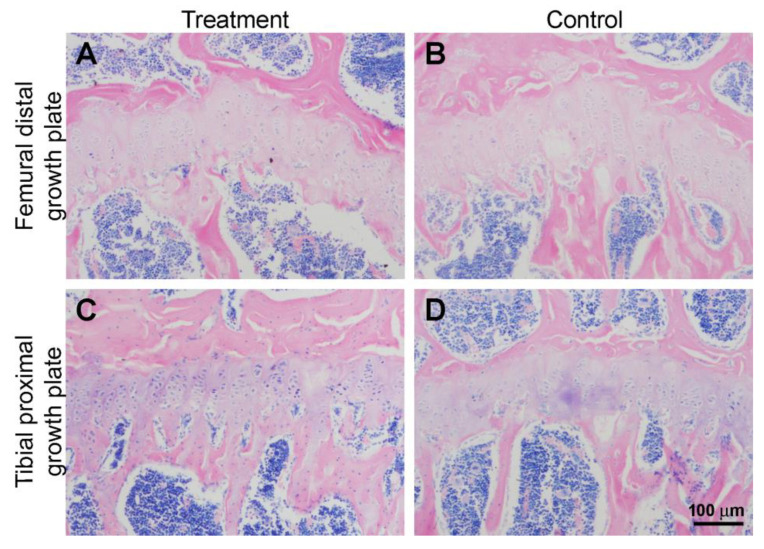
Representative histological sections of the growth plate in the femoral distal metaphysis (**A**,**B**) and tibial proximal metaphysis (**C**,**D**) of treatment and control groups.

Furthermore, the morphological observation of the growth plate in the femoral distal metaphysis and tibial proximal metaphysis for each group (Figure 5) shows that the chondrocytes in the treatment group present the same morphology and typical columnar organization as the control one. 

Histomorphometric results concerning the thickness of the growth plate are shown in Table 3; no significant differences were found between the treatment and control groups.

**Figure 6:**
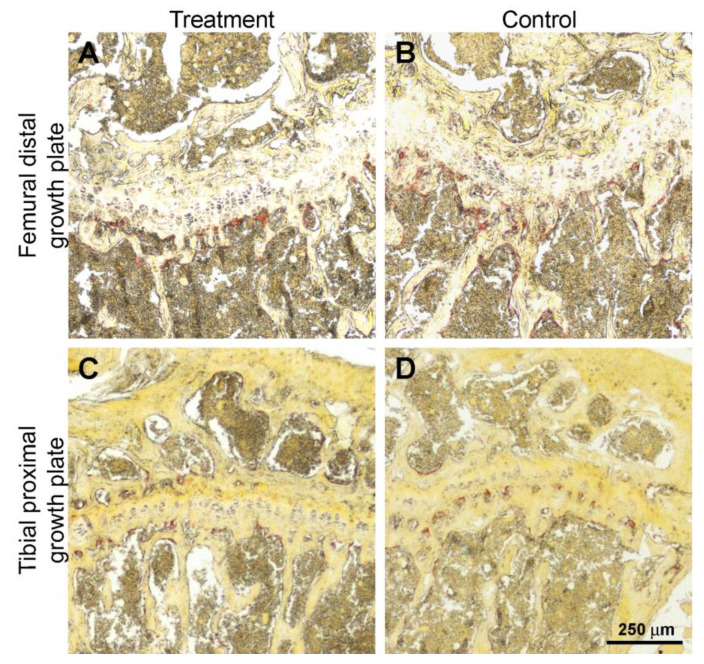
Representative histological sections of the TRAP reaction (osteoclasts are stained in red) in the osteo-chondral interface along the growth cartilage and diaphyseal bone of femoral distal metaphysis (**A**,**B**) and tibial proximal metaphysis (**C**,**D**) of treatment and control groups.

The osteoclasts present in the osteo-chondral interface along the growth cartilage and diaphyseal bone of femoral distal and tibial proximal metaphyseal sections are highlighted by the TRAP reaction as shown in Figure 6. 

The number of osteoclasts (NOc/BS) doesn’t change between treatment and control groups, both in the tibia and the femur, as reported in Table 4.

## 4. Discussion

The findings of this study reveal some unusual responses of bone in 8-week-old C57BL/6 mice subjected to a Mg-deprived diet for 2 months, since unexpectedly the amount of bone didn’t change between Mg-deprived diet mice and control ones, while previous literature data [10,11,12,13,14,15], with special regard to C57BL/6J mice [27], mainly show a reduction in trabecular bone in rodents fed a Mg-deprived diet.

All the animals gained weight during the 8-week study, irrespective of whether they were assigned to the control group (normal diet) or the treated group (Mg-deprived diet). However, the percentage of weight gain at 4 and 8 weeks was significantly lower for treated mice compared with matched controls. The weight difference occurred despite the equal consumption of food by the two groups, in line with the results previously obtained from other groups [11,14,30]. 

In our experimental setting, we observed a 20% reduction in serum Mg concentration in mice receiving the Mg-depleted diet compared with controls. This reduction is less severe than the reduction observed by others that fed C57BL/6J male mice with a low-Mg diet [27,30]. Although a direct comparison of these data might be not appropriate due to differences in the experimental setting (i.e., mice age, treatment time, diet composition), an inverse relationship between Mg serum concentration and both the treatment time and the age of mice at the beginning of the experiment seems to exist.

The adaptive response to Mg status changes is a complex and tightly regulated process that involves, among other mechanisms, the transcriptional and translational control of Mg-membrane transporters. TRPM6/TRPM7 Mg channels mediate the luminal Mg uptake from the pro-urine in the distal convoluted tubule of the kidney [31]. Increased mRNA gene expression of these genes following Mg-dietary restriction has been regarded as a compensatory mechanism to cope with a low-Mg state [32,33]. In our experimental model, we found that the expression of *TRPM7* and *TRPM6* is not significantly altered in the treated group compared with controls. However, the levels of *TRPM6* had a trend toward increasing without reaching statistical significance, possibly due to the small size of the experimental groups. Similarly to *TRPM6*, the expression of *MagT1*, an oxidoreductase that is involved in the trafficking and stability of Mg channels and transporters [34] slightly increased following 8 weeks of Mg deprivation. Overall, these data suggest a good adaptive capacity of 8-week-old C57Bl/6 male mice to long-term dietary Mg deprivation. Interestingly, TRPM6 was included in a group of kidney-specific magnesiotropic proteins involved in the autosomal-recessive disorder hypomagnesemia with secondary hypocalcemia (HSH) [35,36]. 

The histomorphometric results of bone segments investigated in C57BL/6 mice demonstrated that no significant differences were found between the Mg-deprived diet group and the normal-fed diet group for all the parameters that characterize the amount of bone, independently of the architecture analyzed (Table 2). In line with our results, Rude and coworkers [11] didn’t find a significant decrease in trabecular bone in the distal femur after 2 weeks of an Mg-deprived diet in 28-day-old BALB/c female mice; only in the proximal tibia was the loss of trabecular bone significant with respect to control mice. By contrast, Mg deprivation in rats mainly leads to a reduction in bone mass, especially of trabecular bone, even when treatment times are shorter than those used in this study [12,18]. By a micro computed tomography approach, Tu and coworkers demonstrated that the global size of the trabecular bones was smaller in 4-weeks-old C57BL/6J mice fed with Mg-deprived diet for 8 weeks compared with controls [27]. With a similar radiologic approach, Gruber and coworkers [14] showed in 28-day-old BALB/c female mice subjected to a Mg-deprived diet for up to 6 weeks a significant decrease in trabecular bone mineral content in the distal femoral metaphysis versus control mice, while bone mineral density and mean cortical thickness at the femoral midshaft didn’t significantly change between the Mg-deficient mice and control mice. The authors suggested that the different response of bones to a Mg-deprived diet depends on the different bone turnover of trabecular *versus* compact bone; a similar behavior was already observed in our previous investigations in response to a calcium-deprived diet in rats [37,38].

Furthermore, our results on the thickness of the metaphyseal growth cartilage show no differences between the two groups of animals (Table 3); this outcome is in contrast with data reported in the literature. In fact, Rude and coworkers [11] showed that the width of the cartilage growth plate in the proximal metaphysis of the tibia in young BALB/c mice fed a severe Mg-deprived diet for 6 weeks decreased by 33% and the columnar organization of chondrocytes was altered with a decrease in cell proliferation, suggesting that Mg deficiency reduced bone growth. In another animal species, also Gruber and coworkers [39] report that the metaphyseal growth plate in the tibiae of rats subjected to a 6-month dietary restriction of Mg shows significantly reduced formation of chondrocyte columns compared with control animals. 

A third outcome that does not match those present in the literature is the number of osteoclasts calculated in the femoral and tibial metaphyses; in fact, the NOc/BS remains the same independently of the treatment (Table 4). Indeed, it is reported that rats fed a Mg-deprived diet [13,15,18,19] or diets at 10% of the Mg nutritional requirement [12] show in the trabecular bone an increase in the osteoclast number and in the surface covered by osteoclasts, suggesting that the decrease in bone mass is due to an increase in bone resorption. In consideration of the above-discussed findings, it is remarkable to highlight that the results obtained in rats cannot be directly compared with those obtained with mice since these two species show genetic and metabolic differences, and it is not possible to establish reliable general rules to validate extrapolation from one species to another [40,41]. In this regard, it is important to emphasize that Rude and coworkers [11] showed in young BALB/c mice fed an Mg-deprived diet that in the distal femur the number of osteoclasts doesn’t increase and also that BV/TV doesn’t decrease with respect to control mice, thus suggesting that the amount of trabecular bone doesn’t change between the two groups since the NOc/BS remains the same. This result is in line with our finding: in fact, the number of osteoclasts doesn’t change as much as BV/TV between the two groups.

The discrepancy between our results on bone and a few literature data available on mice, in particular those obtained by Tu and coworkers [27] on the C57BL/6J strain, could be due to differences in the methodological approach (histomorphometric vs. radiological) or to the existence of different C57BL/6 substrains that we are not aware of, whose response of bone to Mg deficiency might differ from the expected one. In fact, it has been demonstrated that after many generations of inbreeding, several substrains have been derived from the C57BL/6 founder line [25,42] and are reported to differ in several phenotypes that differentially affect glucose, alcohol, or drug tolerance [43,44,45]. From a genetic perspective, in the same mice strain or probably substrain, Mg dietary restriction acts upon the expression of magnesiotropic and calciotropic genes, inducing adaptation. An essential adaptive role for the distal convoluted tubule in the kidney was described during dietary restriction and subsequent hypomagnesemia since expression levels of selected genes were adjusted [46]. In fact, Liron and colleagues [47] observed sometimes severe variations in the bone phenotype of C57BL/6J mice, hypothesizing that these differences could be attributed to variations among substrains. In particular, they found that C57BL/6J-OLA mice display a significantly lower trabecular bone mass compared with C57BL/6J-RCC and C57BL/6J-JAX substrains. Interestingly, Sankaran and coworkers [48] reported that hind limb unloading in the two substrains of C57BL/6 mice resulted in a different bone response such that bone loss was significantly greater in C57BL/6N mice than in C57BL/6J mice, suggesting that this outcome may be modulated, in part, by the *Herc2*, *Myo18b*, and *Acan* genes. Accordingly, Rendina-Ruedy and colleagues [49] showed that during a high-fat diet C57BL/6J mice were protected against bone loss compared withwith C57BL/6N mice, and therefore they differ in their metabolic and skeletal response. 

By PCR amplification of the *nicotinamide nucleotide transhydrogenase* (*Nnt*) and *a-synuclein* (*SNCA*) genes, we ruled out that the C57BL/6 mice we used for the present study can be traced back to the C57BL/6N or the C57BL/6J-OLA substrains (data not shown). Nonetheless, some substrains’ features have not yet been well identified by biomedical researchers [25] and might develop compensatory mechanisms involving bone and kidney metabolism. Therefore, we cannot exclude that after many multiple generations of inbreeding in our animal facility, new genetic differences, currently uncharacterized, may have developed and contributed to the peculiar observed phenotype, which evidenced skeletal resistance to dietary magnesium depletion.

## 5. Conclusions

In 8-week-old male C57BL/6 mice, the Mg-deprived diet for 8 weeks resulted in a reduction in body weight gain, serum Mg levels, and a trend toward increased expression in kidney-specific magnesiotropic genes without loss of bone mass compared with the control animals. On the contrary, most data present in the literature show that animals fed a Mg-deprived diet develop bone loss, along with a reduction in body weight and serum Mg levels and an increase in kidney magnesiotropic gene expression. Nonetheless, it is necessary to underline that our results cannot be directly compared with those found in the literature. In fact, literature data mainly reported studies involving rats or mice strains different from C57BL/6 mice. The animals’ ages also differed, as did the diet formulations and the treatment time, and all these parameters were expected to influence the outcome. 

The comparison of our results with those obtained in different experimental settings suggests that Mg deprivation produces more consistent and reliable effects on bone resorption in rats than in mice. These differences are likely supported by anatomical differences between the two species that make detecting slight differences at the histological level in small bones more difficult. Moreover, our results and those obtained by others highlight that the widely used C57BL/6 seems to adapt quickly and efficiently to Mg deprivation by adjusting the levels of genes involved in Mg transport through the kidney distal convolute tube. Indeed, another study aimed to investigate the effect of Mg deprivation in C57BL/6J mice on muscle dysfunction and found no detectable variation in muscle fiber morphometry concomitantly with the up-regulation of magnesiotropic genes, including *TRPM6* [30].

We are confident that our results may contribute to the development of roadmaps to facilitate researchers engaged in the study of the effects of Mg deprivation on bones in choosing the right experimental model depending on both their scientific question and methodological approach.

In addition, we suggest the hypothesis that the lack of effects of Mg deprivation on bone could be attributed to genetic differences, which are currently unknown, that may have developed after multiple generations of inbreeding of the C57BL/6J strain in our animal facility. 

In conclusion, it is interesting to consider that certain genetic profiles could provide peculiar skeletal resistance to severe magnesium deficiency, which makes the forthcoming investigations to identify possible genes involved in such resistance particularly intriguing. 

## Figures and Tables

**Table 1:** Sequences of the primers used for qPCR.

Gene	Forward Primer Sequence	Reverse Primer Sequence
*MagT1*	5′-CCAAAGGGGCTGATACATA-3	5′-ATAGAAGAACGATGTGTG-3′
*TRPM6*	5′-AAAGCCATGCGAGTTATCAGC-3′	5′-CTTCACAATGAAAACCTGCCC-3′
*TRPM7*	5′-GGTTCCTCCTCCTGTGGTGCCTT-3′	5′-CCCCATGTCGTCTCTGTCGT-3′
*GAPDH*	5′-TCAAGCTCATTTCCTGGTAT-3′	5′- GTCCAGGGTTTCTTACTCCT-3′

**Table 2:** Histomorphometric parameters measured in L5 vertebral body, femoral distal metaphysis, and tibial proximal metaphysis sections. BV/TV: trabecular bone volume (%); Tb.Th: trabecular thickness (µm); Tb.N: trabecular number; Tb.Sp: trabecular separation (µm); Ct.Th: cortical bone thickness (µm). All values are expressed as mean ± S.D. (n = 7 for each group).

**L5 Vertebral Body**	**Treatment**	**Control**
BV/TV	13.7 ± 3.3	14.5 ± 4.2
Tb. Th	34.2 ± 5.1	38.8 ± 6.1
Tb. N	4.1 ± 1	3.7 ± 0.6
Tb. S	225 ± 67	237 ± 52
Ct Th	68 ± 14.2	60 ± 6.1
**Femoral distal metaphysis**		
BV/TV	13.7 ± 3.8	13.7 ± 3.5
Tb. Th	31.7 ± 6.9	31.5 ± 5.6
Tb. N	4.3 ± 0.8	4.4 ± 0.7
Tb. S	203 ± 45	199 ± 34
**Tibial proximal metaphysis**		
BV/TV	7.3 ± 4.2	7.5 ± 3.3
Tb. Th	26.4 ± 4.8	28.5 ± 4.8
Tb. N	2.68 ± 1.2	2.68 ± 0.7
Tb. S	406 ± 180	378 ± 144

**Table 3:** Thickness (µm) of growth plate measured in femoral distal metaphysis and tibial proximal metaphysis sections. All values are expressed as mean ± S.D. (n = 7 for each group).

Growth Plate Thickness	Treatment	Control
Femoral distal metaphysis	98.5 ± 22.5	102.3 ± 15.7
Tibial proximal metaphysis	106.1 ± 11.6	110.1 ± 23.4

**Table 4:** Number of osteoclasts (NOc/BS) measured in interface along the growth cartilage and diaphyseal bone of femoral distal metaphysis and tibial proximal metaphysis sections. All values are expressed as mean ± S.D. (n = 7 for each group).

NOc/BS	Treatment	Control
Femoral distal metaphysis	9.3 ± 1.9	9 ± 1.9
Tibial proximal metaphysis	8.4 ± 3.7	8.8 ± 3.2

## Data Availability

The data presented in this study are available on reasonable request from the corresponding author.

## Supplementary Materials

The following supporting information can be downloaded at: https://www.mdpi.com/article/10.3390/biomedicines11030655/s1, Table S1:Composition of the experimental diets.
