# Supplementary material for: Unexpected Absence of Skeletal Responses to Dietary Magnesium Depletion: Basis for Future Perspectives?
Source: Biomedicines. 2023 Feb 21;11(3):655. doi: 10.3390/biomedicines11030655 (PMC10045743; doi:10.3390/biomedicines11030655)
Supplement: Supplementary file 1 [file biomedicines-11-00655-s001.zip › biomedicines-2191268-supplementary.pdf]

**Table S1.** Composition of the experimental diets.

| | Mg-Free Diet | Control Diet |
|---|---|---|
| **Contents (%)** | | |
| Crude protein | 15.8 | 19.2 |
| Crude fat | 7.0 | 4.1 |
| Crude fibre | 5.0 | 6.1 |
| Crude ash | 3.6 | 5.9 |
| Carbohydrates | 66.8 | 45.0 |
| Calcium | 0.6 | 0.6 |
| Magnesium | ------------- | 0.2 |
| Phosphorous | 0.5 | 0.5 |
| Sodium | 0.2 | 0.2 |
| Potassium | 0.6 | 0.9 |
| Lysine | 1.4 | 8.1 |
| Methionine | 0.7 | 2.7 |
| Met+Cys | 1.1 | 5.9 |
| **Energy (kcal/kg)** | 3930 | 3227 |
| Kcal% Protein | 16 | 24 |
| Kcal% Fat | 16 | 11 |
| Kcal% Carbohydrates | 68 | 65 |
